# Construction of a Novel Infectious Clone of Recombinant Herpesvirus of Turkey Fc-126 Expressing VP2 of IBDV

**DOI:** 10.3390/vaccines10091391

**Published:** 2022-08-25

**Authors:** Abid Ullah Shah, Zhisheng Wang, Yating Zheng, Rongli Guo, Saisai Chen, Mengwei Xu, Chuanjian Zhang, Yamei Liu, Jichun Wang

**Affiliations:** 1 National Research Center of Engineering and Technology for Veterinary Biologicals/Institute of Veterinary Immunology and Engineering, Jiangsu Academy of Agricultural Sciences, Nanjing 210014, China; 2 Jiangsu Co-Innovation Center for Prevention and Control of Important Animal, Infectious Diseases and Zoonoses, Yangzhou 225009, China; 3 Jiangsu Key Laboratory of Food Quality and Safety, State Key Laboratory Cultivation Base of MOST, Jiangsu Academy of Agricultural Sciences, Nanjing 210014, China; 4 Institute of Veterinary Medicine, Jiangsu Academy of Agricultural Sciences, Nanjing 210014, China

**Keywords:** herpesvirus of turkeys, infectious clone, gC gene, vectored vaccine, VP2, infectious bursal disease virus, field isolates, chicken

## Abstract

The increased virulence of infectious bursal disease virus (IBDV) is a threat to the chicken industry. The construction of novel herpesvirus of turkey-vectored (HVT) vaccines expressing VP2 of virulent IBDV may be a promising vaccine candidate for controlling this serious disease in chickens. We generated a novel infectious clone of HVT Fc-126 by inserting mini-F sequences in lieu of the glycoprotein C (gC) gene. Based on this bacterial artificial chromosome (BAC), a VP2 expression cassette containing the pMCMV IE promoter and a VP2 sequence from the virulent IBDV NJ09 strain was inserted into the noncoding area between the UL55 and UL56 genes to generate the HVT vector VP2 recombinant, named HVT-VP2-09. The recovered vectored mutant HVT-VP2-09 exhibited higher titers (*p* = 0.0202 at 36 h) or similar growth kinetics to the parental virus HVT Fc-126 (*p* = 0.1181 at 48 h and *p* = 0.1296 at 64 h). The high reactivation ability and strong expression of VP2 by HVT-VP2-09 in chicken embryo fibroblasts (CEFs) were confirmed by indirect immunofluorescence (IFA) and Western blotting. The AGP antibodies against IBDV were detected beginning at 3 weeks post-inoculation (P.I.) of HVT-VP2-09 in 1-day-old SPF chickens. Seven of ten chickens immunized with HVT-VP2-09 were protected post-challenge (P.C.) with the virulent IBDV NJ09 strain. In contrast, all chickens in the challenge control group showed typical IBD lesions in bursals, and eight of ten died P.C. In this study, we demonstrated that (i) a novel HVT BAC with the whole genome of the Fc-126 strain was obtained with the insertion of mini-F sequences in lieu of the gC gene; (ii) HVT-VP2-09 harboring the VP2 expression cassette from virulent IBDV exhibited in vitro growth properties similar to those of the parental HVT virus in CEF cells; and (iii) HVT-VP2-09 can provide efficient protection against the IBDV NJ09 strain.

## 1. Introduction

Herpesvirus of turkeys (HVT) is Gallid herpesvirus 1 or nonpathogenic Marek’s disease virus (MDV) (serotype 3) originally isolated from domestic turkeys and widely used as a vaccine to prevent Marek’s disease (MD) in commercial poultry [1,2]. To date, HVT has been used as a potential recombinant vector for other poultry infections, such as Newcastle disease and avian influenza [3,4,5,6]. HVT vectors are promising avian vaccine vehicles for several reasons. HVT has a large genome with multiple nonessential and noncoding regions that are suitable for the insertion of multiple or large antigens [7]. HVT induces both humoral and cellular immune responses and provides long-term protection against pathogens induced in chickens [8].

Infectious bursal disease (IBD) causes long-lasting immunosuppression in poultry [9,10,11], resulting in tremendous economic losses due to vaccine failures and increased susceptibility to opportunistic pathogens [12]. The pathogen of IBD is the infectious bursal disease virus (IBDV), which is highly resistant to many disinfectants and is very difficult to remove from contaminated poultry premises. IBDV encodes five proteins known as VP1, VP2, VP3, VP4, and VP5 [13]. The VP2 protein is considered a major protective antigen that elicits neutralizing antibodies to protect chickens from virulent IBDV [14]. Therefore, the VP2 protein is often used as an immunogenicity antigen for recombinant genetically engineered vaccines [15,16,17,18,19].

HVT is already used as a vector for expressing VP2 of IBDV and other protective antigens of avian pathogens, such as Newcastle disease virus (NDV) [5], Eimeria acervuline [20], avian influenza virus (AIV) [21,22,23], and infectious laryngotracheitis virus (ILTV) [24]. Such recombinant HVT-based vaccines confer excellent and long-lasting simultaneous protective immunity in chickens against pathogens [4,25]. However, most of these HVT recombinants were constructed through conventional homologous recombination techniques, which are extremely time consuming and labor intensive. It is difficult to recover purified recombinant viruses through plaque selection methods [26]. The establishment of herpesvirus as a bacterial artificial chromosome (BAC) [27,28] and its related *EN PASSANT* protocol has facilitated the generation of herpesvirus vector vaccines [29,30]. However, most alpha herpesviruses can grow in cell culture in the absence of various glycoproteins [31]. This differential requirement of glycoproteins is attributed to the strict cell-associated nature. It has been proven that the deletion of glycoprotein B (gB) interferes with the cell-to-cell spread of HVT [32]. However, glycoprotein C (gC) is a nonessential gene, and its deletion does not interfere with the rescue of recombinant HVT. The recombinant virus (rHVT-pmpD-N) was recovered from primary chicken embryo fibroblast (CEF) cells by the replacement of the UL44-UL46 gene of HVT [33]. Therefore, gC is a suitable insertion for an exogenous gene.

In this study, we generated a novel infectious clone of the HVT Fc-126 strain by the insertion of miniF sequences in lieu of the gC gene, and based on the clone, recombinant HVT-VP2-09 was constructed as a bivalent vaccine vector by insertion of the expression cassette of the VP2 gene of a very virulent IBDV field isolate NJ09 strain. The expression of heterologous VP2 by HVT-VP2-09 was demonstrated in CEF cells. We also demonstrated that the HVT-VP2-09 bivalent vaccine administered to one-day-old chicks induced VP2-specific antibodies and conferred protection in vaccinated chicks subsequently challenged with IBDV.

## 2. Materials and Methods

The experiment was approved by the Institutional Animal Care and Use Committee at the Jiangsu Academy of Agriculture Sciences and was performed strictly according to the guidelines provided by the Institutional Biosafety Committee.

### 2.1. Viruses and Plasmids

IBDV (NJ09) was identified and preserved in our lab. The BAC transfer vector plasmid was kindly provided by Professor Nikolaus Osterrieder from the Free University of Berlin [34]. The chicken embryo fibroblasts (CEFs) cells were prepared from SPF chicken embryo eggs from Beijing Merial Vital Laboratories Animal Technology Company Limited and prepared by standard method. All HVT strains were propagated on primary or secondary CEFs. Virus stocks were prepared from CEF cultures, which were infected with viruses at a multiplicity of infection (MOI) of 0.01 and cultured for 72 h in CEFs. PFU titers were determined on CEFs according to the standard titration method [35].

The VP2 expression cassette containing a pMCMV IE promoter and VP2 gene of IBDV was cloned into T-Vector pMD19 (Takara, Japan) with slight modification to generate the plasmid pHVT-VP2-09 (at the UL55-UL56 region). The promoter pMCMV IE included a sequence complementary to the sequence between sites 184,336 and 182,946 in the MCMV genome (GenBank: GU305914.1) followed by a Kozak sequence. The plasmid pHVT-VP2-09-KAN containing the VP2 expression cassette and a kanamycin resistance gene inserted at the *Eco*R I restriction site was constructed by cutting and ligating for further *En Passant* recombination [36].

### 2.2. Cells, Viral DNA Extraction, and Transfection

CEFs were propagated in Earle’s minimal essential medium (EMEM; Gibco, Los Angeles, CA, USA) supplemented with 10% newborn calf serum (NBCS; Gibco, Los Angeles, CA, USA), 100 U/mL penicillin, and 100 μg/mL streptomycin at 37 °C under a 5% CO_2_ atmosphere. Viral DNA was purified from infected cells by sodium dodecyl sulfate (SDS)-proteinase K extraction described previously [37]. Transfection of DNA from plasmids, viruses, or BACs was achieved by calcium phosphate precipitation [38]. Briefly, approximately 200 ng DNA was mixed with water, and then 62 μL of 2 M CaCl_2_ was added dropwise to a total volume of 500 μL. The transfection mixture was incubated overnight at 4 °C followed by adding 500 μL cold 2 × HEPES-buffered saline (HBS) solution dropwise. The medium was replaced with 500 μL of fresh EMEM without NBCS or antibiotics and incubated with the transfection mixture at 37 °C for 3–4 h. Media were discarded, and washed twice with PBS. Then, 1.5 mL of 15% glycerol HBS solution was added, and incubated at 37 °C for 2 min. The transfection solution was replaced with EMEM supplemented with 10% NBCS and antibiotics after washing twice with PBS for culture at 37 °C in an incubator with 5% CO_2_.

### 2.3. Construction of an Infectious Clone of the HVT Fc-126 Strain

The HVT Fc-126 infectious clone was generated with the method modified from that used for the generation of BAC [32]. Briefly, cotransfection of DNA of HVT Fc-126 and pUC19-H1-H2-miniF was conducted on primary CEFs (24 h) to allow for insertion of mini-F sequences in lieu of glycoprotein C (gC). After green plaques were observed under UV light (488 nm), a homogeneous population of mini-F recombinant HVT-miniF was obtained by 6 rounds of picking and plating on CEF cultures in a medium containing mycophenolic acid, xanthine, and hypoxanthine as described previously [34]. Genomic DNA extracted from these HVT-miniF CEF cultures was electroporated into *E. coli* DH10B competent cells (Invitrogen). Positive clones with chloramphenicol resistance were examined through RFLP with *Eco*R I and *Bam*H I to select a clone of the HVT complete genome. The infectivity of HVT-BAC DNA was tested after transfection into CEFs using Lipofectamine 3000 (Invitrogen).

### 2.4. Cloning of the IBDV VP2 Gene and VP2 Cassette

The gene cDNA corresponding to the IBDV-NJ09 VP_2_ open reading frame (1359 nucleotides) was PCR-amplified as described previously [36], by using specific primers (Table 1). Briefly, the PCR products of cDNA VP_2_ were digested with *Hind* III and *Sal* I and cloned into the pMCMV IE vector (Clontech, Tokyo, Japan). The VP_2_ cassette containing the MCMV IE promoter and the SV40 polyadenylation signal was PCR-amplified using primers containing homologous sequences of the HVT UL55-UL56 insertion site.

### 2.5. Construction of HVT BAC with Insertion of VP2 Expression Cassette

**Figure 1:**
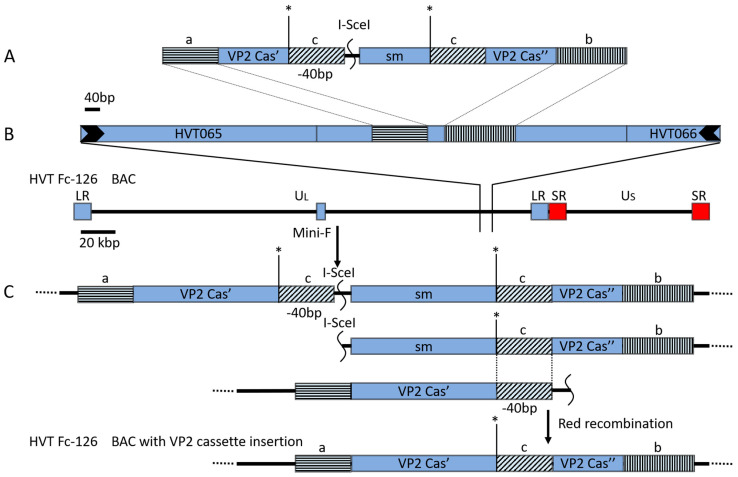
Construction schematic diagram of HVT BAC with insertion of the VP2 expression cassette and RFLP of BACHVT-G, BAC^HVT-VP2-09-KAN^, and BAC^HVT-VP2-09^: (**A**): Kanamycin resistance gene (sm) with a 40 bp homologous sequence (c) was inserted into the Sac I restriction site (*) in the VP2 expression cassette. (**B**): VP2 expression cassette with the selective marker (sm) was inserted into the noncoding area between HVT UL55 and HVT UL56 through the first recombination to generate a recombinant BAC^HVT-VP2-09-KAN^ with both chloramphenicol and kanamycin resistance. (**C**): The second recombination was performed to delete the kanamycin resistance gene to generate the final recombinant VP2 vectored BAC^HVT-VP2-09^ clone. Rectangles with the same type of shading and the same color indicate identical sequences. Scales in bp or kbp are provided.

The VP2 expression cassette was inserted into the noncoding area between UL55 and UL56 in the pHVT-BAC genome through the *En Passant* method (Figure 1). Briefly, PCR was performed using plasmid pHVT-VP2-09-KAN in DNA as a template and a pair of primers (HVT ins VP2 casse F and HVT VP2 casse UL55 R) to amplify the HA cassette with 40 bp homologous sequences flanking both terminals. After digestion with *Dpn* I to eliminate possible plasmid contamination, the PCR product was electroporated via the first red recombination method [36] into competent pHVT BAC cells to generate the first recombination with the cassette at the indicated sites. The target recombinant pHVT-VP2-09 BAC was generated by deletion of the kanamycin resistance gene by the second recombination (Figure 1C). To insert a kanamycin resistance gene into plasmid pHVT-VP2-09, a pair of specific primers (Kan ins’ VP2 F and Kan ins’ VP2 R) (Table 1) were designed with the help of VectorNTI and prime 3 software with two *Sa*c I restriction sites added to both terminals for cutting and ligation. The construct was examined by gene sequencing to check the correct insertion of the kanamycin resistance gene. Another pair of primers (HVT ins VP_2_ casse UL55 F and HVT ins VP_2_ casse UL55 R) (Table 1) was used to insert the VP_2_ cassette into the HVT BAC clone through the *En Passant* protocol. To repair the gC genes of the gC-negative virus, a pair of primers (HVT gC flanking F and HVT gC flanking R) (Table 1) were used to amplify a fragment that included the gC gene and two homologous 1 kbp flanking sequences of gC. The BACs and mutants were subjected to RFLP analysis with *Eco*R I.

### 2.6. Transfection and Isolation of Recombinant rHVT-VP2-09

The recombinant rHVT-VP2-09 viruses were rescued as described previously [34]. Briefly, primary CEFs cultured for 24 h at 37 °C and 5% CO_2_ were cotransfected with 1 mg of pUC19-GC and 5 mg pHVT-VP2-09 DNA using liposomes [34]. To purify the recombinant rHVT-VP2-09 recovering GC fragments, virus-containing cells were passaged for six rounds until the recombinant viruses with cytopathic effect (CPE) express a green fluorescent protein.

### 2.7. One-Step Growth Kinetics

The growth characteristics of viruses were tested on primary or secondary CEFs with an MOI of 0.01, as described previously [39], with a slight modification. Briefly, the virus titers of the cell-associated viruses were checked at 16, 24, 36, 48, 60, 72, 84, and 96 h post-infection (h.p.i.) for parental viruses and mutants. Virus titers were tested following the standard plaque-forming unit (pfu) titration method [34]. The growth kinetics curve was established based on data from three independent experiments [39].

### 2.8. Indirect Immunofluorescence Assay (IFA) and Western Blot Analysis

For the IFA test, chicken embryo fibroblast monolayers (80–90% confluent) were infected with HVT-VP2-09 or HVT, and washed 3 times with phosphate-buffered saline (PBS) and fixed with ice-cold ethanol for 15 min following the appearance of CPE. The cells were overlaid with polyclonal rabbit antibodies produced by vaccination with the IBDV VP2 protein (1:200) and incubated at 37 °C for 1 h. Washed 3 times with PBS and incubated with goat anti-rabbit IgG-FITC antibody (1:1000) (Abcam) at 37 °C for 1 h. The wells were then washed, dried, and analyzed by inversion fluorescence microscopy (Zeiss, SM-33TCI).

For Western-blot analysis, CEFs were infected with HVT-VP2-09 viruses at an MOI of 0.01. Infected cells were lysed with radio immunoprecipitation assay (RIPA) lysis buffer (Biosharp Life Sciences, Beijing, China) and 1% phenyl methyl sulfonyl fluoride (PMSF) protease inhibitor (Solarbio, Beijing, China) for 5 min on ice, and cell lysates were denatured by heating at 95 °C for 10 min. Proteins were separated by SDS-10% polyacrylamide gel electrophoresis (PAGE) and then transferred to nitrocellulose membranes (Merck) as described previously [39]. A mixture of VP2 polyclonal rabbit antibodies was used as the primary antibody for Western blotting, and a 1:10,000 dilution of goat anti-rabbit IgG (Abcam) was used as the secondary antibody. HVT-infected CEFs were used as a control. Samples were detected with enhanced chemiluminescence (Sigma-Aldrich) [40].

### 2.9. Immunization Experiments

For the IBDV protection test, 40 one-day-old SPF chickens were randomly divided into four groups (Table 2). Chickens in group A were inoculated subcutaneously with HVT Fc-126 virus, and chickens in group B were inoculated subcutaneously with HVT-VP2-09 at a dose of 3000 PFU. Chickens in groups C and D were inoculated subcutaneously with 0.2 mL of PBS as controls. After inoculation, sera were sequentially collected from each animal, and antibody titers were determined by the AGP test. Seven weeks after inoculation, all chickens were challenged with the very virulent IBDV field strain NJ09 at a dose of 100 LD_50_ and observed daily for 84 h to analyze morbidity and mortality.

### 2.10. Statistics

Differences in multistep growth kinetics titers of chickens vaccinated with HVT Fc-126 and HVT-VP2-09 were determined by plaque forming units in three independent experiments. Average titers were determined at the indicated time points after infection with an MOI of 0.01. Error bars represent the standard deviations. The time point was determined with a two-way ANOVA with a Tukey post hoc test.

## 3. Results and Discussion

### 3.1. Generation of a Recombinant HVT Fc-126 Strain Harboring Mini-F in Lieu of the gC Gene

**Figure 2:**
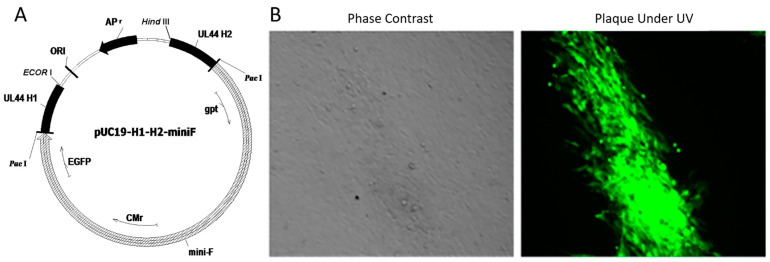
pUC19-H1-H2-miniF and plaques of HVT-miniF: (**A**): Predicted map of the transfer vector pUC19-H1-H2-miniF containing the homologous fragments flanking the gC site. (**B**): A plaque phase contrast is shown under no-UV excitation in the left panel, and plaque phase under UV excitation is shown in the right panel (Green).

First, the transferring vector pUC19-H1-H2-miniF was successfully constructed, containing homologous fragments flanking the glycoprotein C (gC) (UL44) site (Figure 2A). Green plaques under UV light (488 nm) (Figure 2B) were observed at 72–96 h after passaging of the cell culture after transfection of the mixture of approximately 1 μg DNA of the HVT Fc-126 virus genome and approximately 5 μg DNA of the pUC19(HVT)-H1-H2-miniF recombinant transferring vector. The successful construct and expression of HVT-miniF were confirmed by phase contrast and further verified by green fluorescence under UV light because of the green fluorescence protein (GFP) in the vector. The recombinant HVT Fc-126 harboring the mini-F sequence, labeled as HVT-miniF, was selected in a medium containing mycophenolic acid, xanthine, and hypoxanthine.

### 3.2. Generation of Bacterial Artificial Chromosomes of the HVT Fc-126 Strain

**Figure 3:**
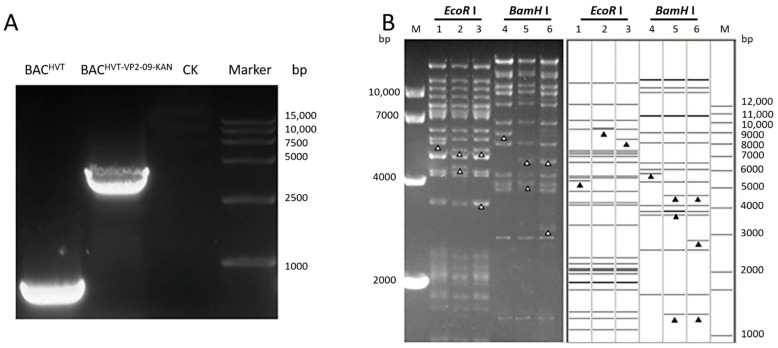
PCR and RFLP of BAC HVT-G, BAC^HVT-VP2-09-KAN^, and BAC^HVT-VP2-09^: (**A**): PCR analysis was performed on the DNA of BAC^-HVT^ and BAC^-HVT-VP2-09^ in the presence of the VP2 expression cassette with primers (HVT UL55 Flanking F/R). (**B**): DNA of BAC^HVT-G^ BAC clone Sa and recombinant BAC^HVT-VP2-09-KAN^ and BAC^HVT-VP2-09^ was prepared by mini-prep and digested with *Bam*H I and *Eco*R I, respectively. Separation of digests was performed by 0.8% agarose gel electrophoresis for 16 h under 40 V. BAC^HVT-G^ is labeled Line 1 and Line 4, which digested with *Eco*R I or *Bam*H I. BAC^HVT-VP2-09-KAN^ is labeled Line 2 and Line 5, which digested with *Eco*R I or *Bam*H I. BAC^HVT-VP2-09^ is labeled Line 3 and Line 6 which digested with *Eco*R I or *Bam*H I. The arrowhead (lane 1) shows a band, while the two arrows (lane 2) show two new bands when digested with *Eco*R I. Meanwhile, the arrow (lane 3) shows a new band and the same arrow as Lane 2 when digested with *Eco*R I. Lanes 4–6 are the *Bam*H I digestion patterns of BAC^HVT-G^ and BAC^HVT-VP2-09-KAN^ and BAC^HVT-VP2-09^, respectively. Their results are the same as expected. The prediction of digestion was performed using the HVT whole genome sequences as a reference (GenBank ID: AF291866). Predicted RFLP patterns of BAC^HVT-VP2-09-KAN^ and BAC^HVT-VP2-09^ compared to the BAC^HVT-G^ clone with size markers from 1 to 12 kb on the right. M: DL 10,000 DNA Marker (Takara).

Colonies with resistance to chloramphenicol were obtained after electroporating DNA of HVT Fc-126 harboring mini-F sequences into *E. coli* DH10B competent cells. Then, the Midi-prep DNA of the selected colonies was electroporated into *E. coli* GS1783 competent cells to generate the infectious clone BAC^HVT-G^ for further VP2 cassette insertion with the *En Passant* method. RFLP of BAC^HVT-G^ with *Bam*H I and *Eco*R I showed suspected patterns compared to in silico predictions based on the published whole genome of the HVT Fc-126 strain (GebBank: AF291866) (Figure 3B).

### 3.3. Construction of the Recombinant HVT BAC with the Expression Cassette of IBDV VP2

Following the *En Passant* protocol, we sought to insert the expression cassette of VP2 from IBDV (NJ09) (Figure 1). The insertion was targeted in lieu of the gC gene of the HVT genome. With the first Red recombination, approximately 300 ng of PCR product of the expression cassette of VP2 was gel-purified with a DNA gel purification kit (Shanghai Generay Biotech Co., Ltd., Shanghai, China) and electroporated into BAC^HVT-G^. Several colonies resistant to kanamycin and chloramphenicol were obtained 2 days after plating. One colony terminated BAC^HVT-VP2-09 K+^ was selected for further deletion of the kanamycin resistance marker gene by the 2nd Red recombination, resulting in colonies with resistance to chloramphenicol but not kanamycin. One of the final recombinant BACs containing the VP2 expression cassette, termed BAC^HVT-VP2-09^, was selected for PCR and RFLP analysis. PCR results showed bands of 450 bp and 3757 bp for BAC^HVT^ and BAC^HVT-VP2^, respectively. These results were exactly the same as suspected, and subsequent sequencing proved the correct insertion (Figure 3A). RFLP patterns corresponded well with the predicted patterns after digestion with *Bam*H I and *Eco*R I (Figure 3B). After digestion with *Eco*R I, BAC^HVT-VP2-09-KAN^ missed a band of approximately 5500 bp observed in BAC HVT-G; instead, two bands of approximately 5000 bp and 4600 bp were present, indicating the correct insertion of the VP2 expression cassette. BAC^HVT-VP2-09^ missed a band of approximately 4600 bp observed in BAC^HVT-VP2-09-KAN^, and instead, a band of approximately 3600 bp was present, indicating the deletion of the selection marker. After digestion with *Bam*H I, BAC^HVT-VP2-09-KAN^ missed a band of approximately 6000 bp observed in BAC HVT-G, and instead, three bands of approximately 4600 bp, 3900 bp, and 1600 bp (data not shown) were present, indicating the correct insertion of the VP2 expression cassette. BAC^HVT-VP2-09^ missed a band of approximately 3900 bp observed in BAC^HVT-VP2-09-KAN,^ and instead, a band of approximately 2900 bp was present, indicating the deletion of the selection marker (Figure 3B). RFLP, PCR, and sequencing results showed correct insertion of the VP2 expression cassette and deletion of the kanamycin resistance selection marker, even though the RFLP pattern was slightly different from the expected pattern, which might be due to the different passages of HVT Fc-126 used in this study and the reference strain genome.

### 3.4. Rescue of the Recombinant HVT BAC Containing the VP2 Expression Cassette

**Figure 4:**
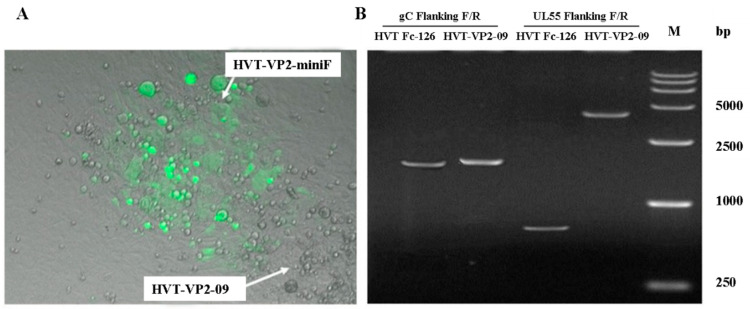
Rescue of the recombinant HVT BAC containing the VP2 expression cassette: (**A**): Rescue plaques were observed under UV light (488 nm). Fluorescent plaques were infected with recombinant HVT-VP2-09-miniF, and nonfluorescent plaques were infected with recombinant HVT-VP2-09 that recovered the gC gene. (**B**): DNA of a PCR fragment amplified with DNA of HVT Fc-126 or HVT-VP2-09 as a template and gC FlankingF/R or UL55 FlankingF/R as primers (Table 1).

A mixture of approximately 1 μg DNA of BAC HVT-VP2-09 (NJ09) and approximately 5 μg DNA of a PCR fragment was amplified with DNA of HVT Fc-126 as a template and with a pair of primers (HVT INS’ gC F/R) was transfected into CEF cells. Nonfluorescent plaques were observed under UV light (488 nm) at 3–4 days post-seeding of the transfection cell culture (Figure 4A). A homogeneous population of purified viruses, termed HVT-VP2-09 (NJ09), was isolated after several rounds of picking and plating. PCR of gC with primers (HVT gC Flanking F/R) and VP2 expression cassette with primers (HVT UL55 Flanking F/R) showed bands of 2059 bp and 3757 bp, respectively, as suspected (Figure 4B). Subsequent sequencing results proved the correct recovery of the gC gene as in the parental virus and confirmed the correct insertion of the VP2 cassette in the noncoding area between UL55 and UL56.

### 3.5. Growth Kinetics of HVT Fc-126 Vectored VP2

**Figure 5:**
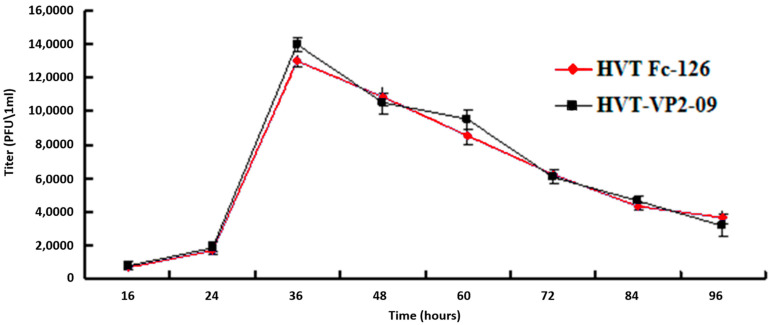
Multistep growth kinetics of HVT-VP2-09 and DEV-H5 (UL55) compared to HVT Fc-126 on CEFs. Titers of cell-associated viruses. Titers were determined by plaque forming units of 0.1 mL of samples in three independent tests. Average titers were determined at the indicated time points after infection with an MOI of 0.01. Error bars represent the standard deviations. The recombinant HVT-VP2 virus titers showed similar growth properties as the parental virus HVT Fc-126 strain (*p* = 0.07 at 72 h and *p* = 0.08 at 84 h).

The growth properties of the engineered mutant (HVT-VP2-09 (NJ09)) were compared with those of the parental virus (HVT Fc-126) in three independent experiments. Virus titers were tested on fresh CEF cells after trypsinization of virus-infected cells without freeze-thawing. The virus titers determined at 0 h, 16 h, 24 h, 36 h, 48 h, 60 h, 72 h, 84 h, and 96 h post-infection with an MOI of 0.01 showed similar growth properties as the parental virus HVT Fc-126 strain (*p* = 0.23 at 36 h and *p* = 0.37 at 48 h) (Figure 5). These results showed that the BAC constructed in this study was the whole genome clone of the HVT Fc-126 strain and the insertion of the VP2 cassette in the noncoding area between UL55 and UL56 did not affect the growth ability of the mutant virus, at least in CEF cells.

### 3.6. Expression of IBDV VP2 by Recombinant HVT Fc-126 Vectored VP2

**Figure 6:**
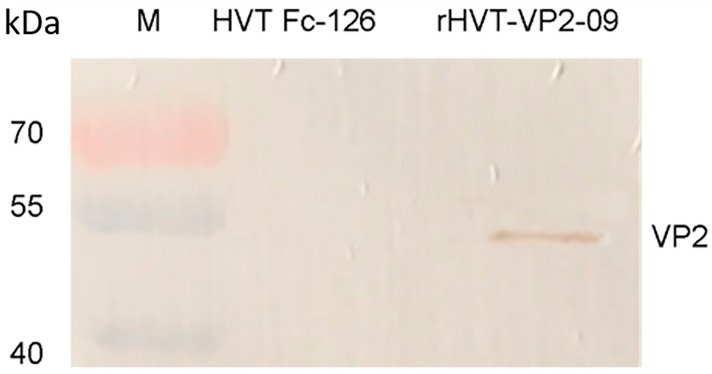
Western-blotting analysis of VP2 expression of HVT-VP2-09. Lysates of CEFs infected with HVT Fc-126 and HVT-VP2-09. Proteins were separated by SDS-10% polyacrylamide gel electrophoresis and transferred to nitrocellulose membranes (Merck). A mixture of VP2 polyclonal rabbit antibodies was used as the primary antibody, and a 1:10,000 dilution of goat anti-rabbit IgG (Abcam) was used as the secondary antibody. Detection was performed with enhanced chemiluminescence (Sigma-Aldrich). Lane M: DNA marker (PageRuler^TM^ Plus, Fermentas).

**Figure 7:**
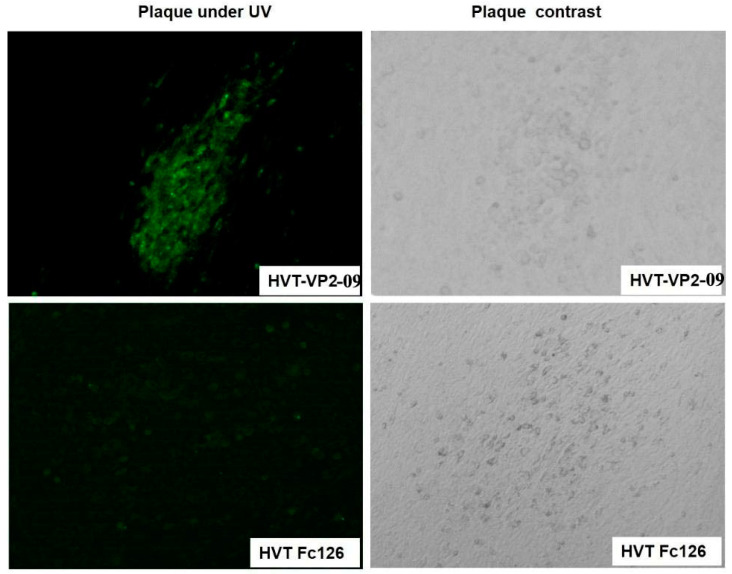
Indirect immunofluorescence assay (IFA). Chicken embryo fibroblast monolayers (80–90% confluent) were infected with HVT-VP2-09 or HVT Fc-126. The wells were overlaid with polyclonal rabbit antibodies produced by vaccination with the IBDV VP2 protein (1:200) and incubated at 37 °C for 1 h. The wells were incubated with anti-rabbit IgG-FITC antibody produced in goat (1:1000) (Abcam) at 37 °C for 1 h. Fluorescent plaques infected with recombinant HVT-VP2-09 were visible by inversion fluorescence microscopy; however, nonfluorescent plaques infected with recombinant HVT Fc-126 were visible by inversion fluorescence microscopy.

In Western blotting, while proteins with molecular masses of approximately 48 kilodalton (KDa) were observed in lysates of CEFs infected with HVT Fc-126 vector VP2, no protein band was detected in CEFs infected with HVT Fc-126 (Figure 6). The sizes of the detected bands were in accordance with the theoretical results we expected. An indirect immunofluorescence test was performed with the first antibody against VP2 of IBDV obtained from rabbits. Strong signals were observed on HVT-VP2-09 (NJ09) plaques using goat anti-rabbit IgG antibodies. In contrast, no signals were observed on parental HVT Fc-126 strain plaques under the same treatment conditions (Figure 7). These results showed that the mutant HVT-VP2-09 (NJ09) could express VP2 with high reactivation and that VP2 expression of HVT-VP2-09 (NJ09) was strong and stable in vitro on CEFs.

### 3.7. Immunogenicity of HVT Fc-126 Vectored VP2 in Chickens

**Figure 8:**
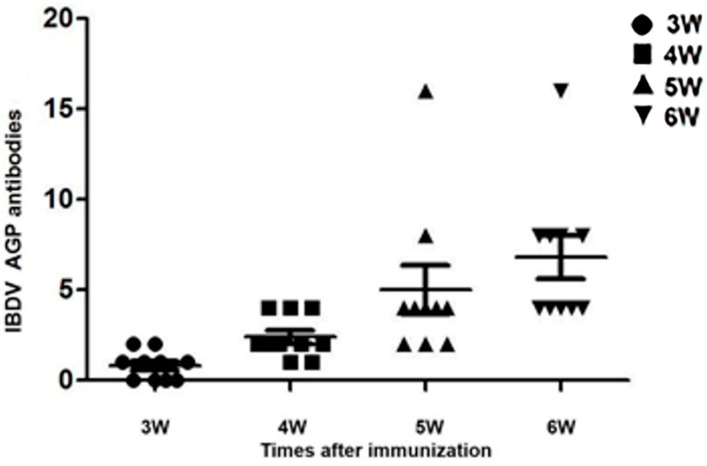
IBDV AGP antibodies after immunization with HVT-VP2-09. The maximum dilution of IBDV AGP antibodies was tested at 3, 4, 5, and 6 weeks after inoculation with HVT-VP2-09. IBDV AGP antibodies were not detected in the other groups (not shown).

All chickens were healthy post-inoculation, and no AGP antibodies against IBDV were detected in chickens in groups A, C, or D. AGP antibodies were detected in the chickens of group B at 21, 28, 35, and 42 days post-inoculation (Figure 8).

All chickens other than group D were challenged with virulent NJ09 at seven weeks post-inoculation and observed daily for 84 h. At 84 h post-challenge (P.C.), eight chickens died in group C, and for chickens in group A, a total of seven chickens died in the 84 h P.C. Meanwhile, no chickens died in group B P.C. (Table 2). All dead chickens showed edema in bursals. No chickens died in group D. All surviving chickens were euthanized to check lesions in bursals and muscles. All the chickens in groups A and C showed edema in bursals and/or hemorrhage in the keratin between the glandular stomach and gizzard. Three chickens in group B showed edema in bursal.

**Figure 9:**
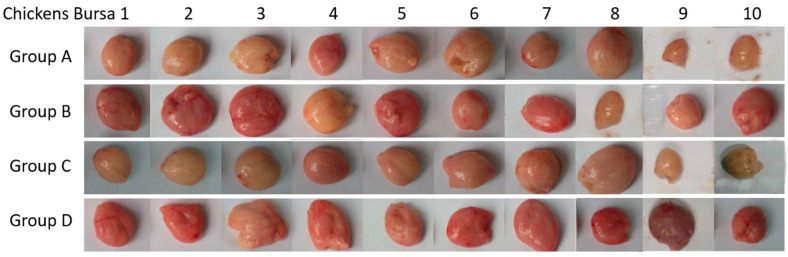
The histologic appearance of the bursa of Fabricius in chicks after IBDV (NJ09) challenge Chickens in group **A** were inoculated subcutaneously with HVT Fc-126 virus, and those in group **B** were inoculated subcutaneously with HVT-VP2-09 at a dose of 3000 PFU. Chickens in groups **C** and **D** were inoculated subcutaneously with 0.2 mL of PBS as controls. Six weeks after inoculation, the chickens of groups A–C were challenged with virulent NJ09 at a dose of 100 LD_50_ and observed daily for 84 h to analyze the histologic appearance changes.

The chickens in group D showed normal bursal and keratin between the glandular stomach and gizzard (data not shown) (Figure 9) (Table 2). These results showed that HVT-VP2-09 (NJ09) could induce efficient protection against virulent IBDV NJ09 strain challenge, indicating the successful construction of the infectious clone of the whole genome of the HVT Fc-126 strain and the promising prospect of HVT-VP2-09 (NJ09) to serve as a vectored vaccine candidate. Further research on the protection against the MDV challenge is needed.

## 4. Conclusions

In this study, a novel bacterial artificial chromosome of the HVT Fc-126 strain was generated with the insertion of mini-F sequences in lieu of the gC gene. The HVT vector HVT-VP2-09 serves as an infectious clone of the HVT Fc-126 strain’s entire genome. In conclusion, the vectored vaccine was successfully constructed using the *En Passant* protocol using BAC with the insertion of a VP2 expression cassette of the highly contagious IBDV field isolate NJ09 strain. Vaccinated 1-day-old SPF chickens can produce significant antibodies as early as the third week and can resist the attack of virulent IBDV strains as late as the sixth week, indicating a very strong immune protection effect. This will be of great significance for the construction of a multivalent turkey herpesvirus live vector vaccine. To our knowledge, the HVT Fc-126 strain has never been used as a vaccine candidate before, and the HVT-VP-09 can be used as a potential vaccine against IBDV strain NJ09 in chicken.

## Figures and Tables

**Table 1:** PCR amplification using specific primers.

Construct	Sequence
KAN INS’ VP2 F	*5′-TTAGAGCTCCTCGCTGCAGGCGGCCGCTCTAGAACTCGTCGA* *TCGCAGCGGGATGACGACGATAAGTAGGGATAAC-3′*
KAN INS’ VP2 R	*5′-CGCGAGCTCGGGTAATGCCAGTGTTACAACCA-3′*
HVT INS VP_2_ CASSE F	*5′-GGGCTATATGTTATTAAATAAAATAATTGACCAGTGAACACTAG* *TGGATCCCCCAACTCC-3* *′*
HVT INS VP_2_ CASSE R	*5′-ATGAATATTTGCAACCAATGCATTGAATAAACTAACATTATTGTC* *GACTCTAGAGGATCCG-3* *′*
HVT gC FLANKING F	*5′-AGCAGCCAGTGTCGTGAAATC-3′*
HVT gC FLANKING R	*5′-TTGGCCGTGAGCTTGATATTCG-3′*
UL55 FLANKING F	*5′-GAATCTATGCCCATATCTGG-3′*
UL55 FLANKING R	*5′-CATGATATCTTATCCAAGCGG-3′*
HVT INS’ gC F	*5′-GGGGATCCACTAGTGAATTT-3′*
HVT INS’ gC R	*5′-CGGATCCTCTAGAGTCGACAA-3′*
VP2 F	*5′-GCGATGACAAACCTGCAAGATCAAAC-3′*
VP2 R	*5′-GATCAAGGTTACCTCCTTATAGC-3′*

**Table 2:** Groups of animals tested to evaluate the efficacy and immunogenicity of HVT-VP2-09.

Groups	Total	Vaccine or PBS	IBDV AGP Antibodies				Challenge (NJ09)	Morbidity	Fatality
			3 Weeks	4 Weeks	5 Weeks	6 Weeks			
A	10	HVT Fc-126 (3000PFU)	0	0	0	0	Yes	10/10	7/10
B	10	HVT-VP2-09 (3000PFU)	0,0,0,0 1,1,1 1,2,2	1,1,2,2 2,2,2 4,4,4	2,2,2,4 4,4,4 4,8,16	4,4,4,4 4,8,8 8,8,16	Yes	3/10	0/10
C	10	PBS	0	0	0	0	Yes	10/10	8/10
D	10	PBS	0	0	0	0	No	0/10	0/10

## Data Availability

Not Applicable.
